# Smoking and risk of sepsis: A secondary analysis of a UK Biobank prospective cohort with protein pathway mediation

**DOI:** 10.18332/tid/226617

**Published:** 2026-10-15

**Authors:** Yangyanqiu Wang, Maomao Cao, Wei Jiang, Li Weng, Bin Du

**Affiliations:** 1 Medical Intensive Care Unit, State Key Laboratory of Complex Severe and Rare Diseases, Peking Union Medical College Hospital, Peking Union Medical College and Chinese Academy of Medical Sciences Beijing China

**Keywords:** smoking, sepsis, immune-inflammatory pathway, mediation

## Abstract

**Introduction:**

Sepsis is a global health concern with high mortality. Although smoking impairs immune function and increases infection risk, its specific association with sepsis remains unclear. This study aimed to examine the association between smoking and sepsis and clarify the potential mediating mechanisms.

**Methods:**

This secondary analysis of the UK Biobank prospective cohort included 480344 participants without baseline sepsis and with complete smoking data. Smoking characteristics were collected via touchscreen questionnaires at baseline. Sepsis cases were identified through linkage to hospital admissions. The association between smoking and sepsis was evaluated using multivariable Cox proportional hazards models. Proteomics data were derived from the UKB Pharma Proteomics Project (2923 unique proteins, Olink), available in 50564 participants. Pathway over-representation analyses were performed to identify biological pathways in participants with and without sepsis. Principal component analyses were performed to extract weighted scores for each pathway. Subsequently, weighted scores were used in mediation analyses to investigate how the pathways mediated the association between smoking and sepsis.

**Results:**

Compared with never smokers, current smokers had significantly higher risks of total sepsis (adjusted hazard ratio, AHR=1.42; 95% CI: 1.39–1.46), pulmonary sepsis (AHR=1.57; 95% CI: 1.48–1.66) and non-pulmonary sepsis (AHR=1.36; 95% CI: 1.30–1.42). Smoking cessation yielded a time-dependent protective effect, with an AHR of 0.73 (95% CI: 0.70–0.76) among individuals who quit smoking for ≥30 years. Pathway enrichment analysis of proteomic profiles identified activated immune-inflammatory cascades in sepsis cases, particularly neutrophil degranulation and extracellular matrix organization. These immune-inflammatory pathways contributed to the smoking–sepsis association, with mediation proportions ranging from 14.4% to 24.7% for pulmonary sepsis and from 25.0% to 69.9% for non-pulmonary sepsis.

**Conclusions:**

Smoking is a significant risk factor for sepsis, and these associations could be mitigated by smoking cessation to some extent. The observed associations might be partially mediated through immune-inflammatory pathway dysregulation.

## Introduction

Sepsis remains a critical global health challenge, with an estimated 166 million cases and 21.4 million deaths worldwide in 2021, accounting for nearly one-third of all global mortality^1^. Despite the development of critical care and supporting techniques, effective treatments remain insufficient due to complex pathophysiological mechanisms of sepsis^2,3^. The substantial burden of sepsis underscores the urgent need to understand and address modifiable risk factors in lifestyle. Smoking is one of the most common modifiable risk factors for numerous diseases. In 2020, an estimated 1.3 billion people worldwide used tobacco, and smoking-related illnesses are estimated to be responsible for approximately 1 billion deaths annually^4^.

Tobacco smoking generally produces harmful compounds (ie, nicotine), which contribute to both immune dysfunction and immunosenescence, thereby potentially predisposing individuals to infections in multiple organ systems, including the pulmonary tract^5,6^. Additionally, smoke-derived free radicals like peroxides and nitric oxide damage cell membranes and DNA, and activate inflammatory pathways such as nuclear factor kappa-light-chain-enhancer of activated B cells (NF-κB) and tumor necrosis factor (TNF) in macrophages and epithelial cells, further compromising vascular endothelial function^7,8^. Together, these changes may increase the risk of sepsis and subsequent organ failure. However, a study conducted in Canada showed that current smokers among patients with bacteremic pneumococcal pneumonia had lower in-hospital mortality and were more often infected with less virulent serotypes^9,10^. Given the complex immunological effects of smoking, its overall impact on sepsis outcomes remains uncertain.

This study aimed to provide evidence substantiating the association of smoking with sepsis, utilizing a prospective cohort of large community-based data from the UK Biobank (UKB)^11,12^. By identifying potential protein signatures and signaling pathways related to sepsis and applying mediation analysis, we seek to clarify whether smoking contributes to the occurrence of sepsis through the regulation of these pathways. The findings are expected to illustrate the understanding of both the epidemiological role of smoking in sepsis development and the underlying pathophysiological mechanisms involved.

## Methods

### Study design and population

This secondary analysis used data from the UKB, a large-scale, population-based prospective cohort study that comprehensively investigates genetic, lifestyle, and environmental determinants of diverse human diseases. The detailed design and data collection procedures of the UKB have been reported previously^11,12^. Briefly, the UKB recruited 502536 participants aged 40–69 years across 22 assessment centers throughout the United Kingdom between 2006 and 2010. Eligible participants were registered with the National Health Service and resided within 25 miles of a study center. Baseline assessments included touchscreen questionnaires, physical and functional examinations, computer-assisted interviews, and blood specimen collection. Longitudinal health outcomes were continuously captured through record linkage with national primary care, hospital inpatient, cancer, and death registry databases. The study protocol was approved by the North West Multi-Centre Research Ethics Committee (reference 21/NW/0157, 18 June 2021), and all participants provided electronically signed informed consent.

Participants were eligible for inclusion if they had no prevalent sepsis at baseline and possessed complete data on smoking status. Of the total UK Biobank cohort (n=502536), we excluded individuals with baseline sepsis (n=17258), missing smoking data (n=2871), missing recruitment date (n=2), and missing racial information (n=1656). Following these inclusion and exclusion criteria, the final analytical sample comprised 480344 participants.

### Variables

#### Exposure (smoking status)

Smoking data were collected during baseline assessment (2006–2010) using two core touchscreen questionnaire questions: ‘Do you smoke tobacco now?’ and ‘In the past, how often have you smoked tobacco?’. Participants were categorized as never smokers (no prior smoking), former smokers (quit before recruitment), or current smokers (occasional/regular smoking at assessment).

Pack-years of smoking were calculated for former and current smokers: (cigarettes per day/20) × (age at cessation - age at initiation) for former smokers, and (cigarettes per day/20) × (age at recruitment - age at initiation) for current smokers. For participants with abstinence >6 months, 0.5 years was subtracted from total smoking duration. Special handling included: missing values for those who started and quit before the age of 16 years; initiation age set to 16 years for those who started before 16 years but quit at/after 16 years; and 0 pack-years for those with identical start/cessation ages and abstinence >6 months. Total smoking exposure was categorized as ≤10, 11–20, 21–30, and >30 pack-years. For former smokers, cessation duration (quitting to baseline) was stratified into ≤10, 11–20, 21–30, and >30 years.

#### Outcomes (sepsis and related events)

The primary outcome was incident sepsis, defined using the International Classification of Diseases, Tenth Revision (ICD-10; Supplementary file Table 1)^13,14^. Secondary outcomes included pulmonary/non-pulmonary sepsis (based on infectious etiology), critical care admission (level 2/3 critical care during sepsis admission), and sepsis-related mortality (death within 28 days of sepsis admission). All-cause mortality was extracted from relevant national death registrations. Relevant critical care data were available for participants in England. Follow-up duration was calculated from recruitment to the earliest of: sepsis diagnosis, all-cause death, loss to follow-up, or administrative censoring. Relevant UKB Field-IDs are listed in Supplementary file Table 2.

#### Covariates

Covariates included age, sex, ethnicity, education level, Townsend Deprivation Index (TDI), body mass index (BMI), drinking status, physical activity (summed metabolic equivalent of task [MET] minutes per week; 1 MET represents the resting metabolic rate, defined as an oxygen consumption of 3.5 mL/kg/min), and comorbidities (hypertension [ICD-10: I10–I13, I15], diabetes [E10–E14], chronic respiratory disease [J43–J46], chronic liver disease [K70–K76], chronic kidney disease [N18], cardiovascular disease [I20–I21, I24–I26, I46, I50, I60–I64, I70–I79], cancers [C3+]), as well as history of surgery and medication use (antihypertensives, lipid-lowering drugs, insulin). Multiple imputation was used for missing covariate data.

### Proteomics

Plasma protein data were derived from the UKB Pharma Proteomics Project (UKB-PPP)^14^. Participants comprised three subgroups: 1) a random baseline subsample of 46595 participants, selected to ensure representativeness of the full cohort; 2) 6376 participants preselected by 13 partner pharmaceutical companies (approximately 500 per company); and 3) 1268 participants from the COVID-19 repeat imaging study. This yielded a total of 54219 participants with available proteomics data.

EDTA plasma samples were collected at the baseline recruitment visits between 2006 and 2010. Protein measurements were performed at Olink (Uppsala, Sweden) between May 2021 and November 2022 using the Olink Explore 3072 platform. Quality control (QC) was performed by UKB according to the following criteria: quantitative success rate >90%; at least 50 samples per assay plate with sufficient sequencing reads; median within-plate and between-plate coefficients of variation (CV) <11% (90th percentile <25%); and median within-plate CV <8%. After QC, a total of 2923 unique proteins across eight Olink panels (cardiometabolic, neurology, inflammation, oncology; two versions each) were available for analysis. Proteins with >20% missing values were excluded, and the remaining 2920 proteins were imputed with median values and normalized using z-scores.

Among the 54219 participants who had proteomic data, we applied the same inclusion and exclusion criteria as the main cohort. Those with baseline sepsis, missing smoking data, missing recruitment date, and missing racial information were excluded, and 50564 participants were ultimately included in the proteomic analysis.

### Statistical analysis

Baseline characteristics were summarized by smoking status: normally distributed continuous variables as mean ± SD, non-normally distributed as median (IQR), and categorical variables as frequency (%). Sepsis incidence rates were calculated per 1000 people. Cox proportional hazards regression models evaluated associations between smoking factors (status, pack-years, cessation duration) and outcomes, with proportional hazards assumptions verified via Schoenfeld residuals (all p>0.05). Trend p-values were calculated using Wald tests for linear contrast. Three adjustment models were used: Model 1 (age, sex, ethnicity, education, TDI); Model 2 (additionally BMI, drinking, physical activity); Model 3 (further adjusted for comorbidities, surgery/medication history). Kaplan-Meier curves compared cumulative sepsis incidence by smoking factors, with significance assessed via the Log-rank test.

Cox models also evaluated associations between plasma protein levels and sepsis, adjusted for age, sex, ethnicity, BMI, TDI, and drinking status. Hazard ratios (HRs) with 95% confidence intervals (CIs) were extracted, and p-values adjusted via Bonferroni correction. Upregulated differential proteins were defined as HR >1 and adjusted p<0.05. Pathway enrichment analysis for upregulated proteins was performed in R using *clusterProfile*r (Kyoto Encyclopedia of Genes and Genomes (KEGG) pathways, v3.22.0) and *ReactomePA* (Reactome pathways, v3.22.0)^15-18^, with significance defined as Benjamini-Hochberg-adjusted p<0.05.

Principal component analysis reduced dimensionality of proteins within each enriched pathway. Weighted pathway scores were derived from the first principal component (capturing maximum variance) and used in causal mediation analyses (CMAverse package, cmest function, v0.1.0)^19^ to explore pathway mediation in the smoking-sepsis relationship. Total effect was decomposed into natural direct and indirect effects. The proportion mediated was calculated as pm = (R_TE_ - R_CDE_)/(R_TE_ - 1) where R_TE_ and R_CDE_ represent the HRs for the total effect and controlled direct effect, respectively. Nonparametric bootstrapping with 1000 replicates was adopted to compute 95% CIs and p-values for mediation estimates. The proportional hazards assumption for mediation models was verified via Schoenfeld residual tests (all p>0.05). Bootstrap with 1000 replicates was used for inference. All statistical analyses were carried out in R v4.5.2. A two-tailed p<0.05 was considered statistically significant.

## Results

### Basic characteristics

The cohort included 480344 participants: never smokers (n=264030), former smokers (n=165877), and current smokers (n=50437). During a median 13-year follow-up, 55061 sepsis cases occurred, with incidence increasing by smoking status: never (9.6%), former (13.2%), current (15.0%); this trend was consistent for pulmonary (1.8%, 2.6%, 3.3%) and non-pulmonary sepsis (5.1%, 6.5%, 6.9%) (Table 1; and Supplementary file Table 3).

**Table 1:** Baseline demographic, clinical, and lifestyle characteristics, by smoking status, among UK Biobank participants free of prevalent sepsis at baseline (N=480344) (recruitment: 2006–2010; median follow-up: 13 years)

*Characteristics*	*Never smokers* *(N=264030)* *n (%)*	*Former smokers* *(N=165877)* *n (%)*	*Current smokers* *(N=50437)* *n (%)*
**Age at entry** (years), mean ± SD	55.80 ± 8.15	58.14 ± 7.70	54.63 ± 8.15
**Age started smoking** (years), mean ± SD		17.26 ± 3.63	17.64 ± 6.16
**Age stopped smoking** (years), mean ± SD		39.21 ± 11.78	
**Sex**			
Men	156035 (59.1)	81822 (49.3)	23080 (45.8)
Women	107995 (40.9)	84055 (50.7)	27357 (54.2)
**Ethnicity**			
White	245870 (93.1)	160896 (97.0)	47251 (93.7)
Mixed	1340 (0.5)	909 (0.6)	518 (1.0)
Asian/Asian British	7218 (2.7)	1236 (0.8)	893 (1.8)
Black/Black British	5407 (2.1)	1316 (0.8)	952 (1.9)
Other	4195 (1.6)	1520 (0.9)	823 (1.6)
**TDI**, median (IQR)	-2.39 (−3.78 – -0.01)	-2.11 (−3.61–0.51)	-0.41 (−2.81–2.78)
**College degree**	95055 (36.0)	49205 (29.7)	11558 (22.9)
**BMI** (kg/m^2^)			
<18.5	1421 (0.5)	513 (0.3)	553 (1.1)
18.5–24.9	93325 (35.4)	46347 (27.9)	17670 (35.0)
25.0–29.9	109822 (41.6)	73730 (44.5)	20927 (41.5)
≥30	59462 (22.5)	45287 (27.3)	11287 (22.4)
**Current drinkers**	239083 (90.6)	156636 (94.4)	46395 (92.0)
**Physical activity** (MET-min/w), median (IQR)	1773 (819–3516)	1824 (831.0–3607.5)	2000 (900–4000)
**Comorbidities**			
Hypertension	93807 (35.5)	73764 (44.5)	19532 (38.7)
Diabetes	20907 (7.9)	18613 (11.2)	5797 (11.5)
Chronic respiratory disease	38937 (14.8)	30956 (18.7)	12642 (25.1)
Chronic liver disease	7888 (3.0)	6610 (4.0)	2651 (5.3)
Chronic renal failure	11772 (4.5)	10196 (6.2)	2457 (4.9)
Cardiovascular diseases	48216 (18.3)	43470 (26.2)	13657 (23.1)
Cancer	48659 (18.4)	37759 (22.8)	10162 (20.2)
**Medications**			
Blood pressure lowering medication	22051 (8.4)	25329 (15.3)	5446 (10.8)
Cholesterol lowering medication	19129 (7.3)	24313 (14.7)	5788 (11.5)
Insulin	1229 (0.5)	1437 (0.8)	434 (0.9)
**Outcomes**			
Sepsis	25346 (9.6)	21963 (13.3)	7562 (15.0)
Pulmonary sepsis	4694 (1.8)	4259 (2.6)	1664 (3.3)
Non-pulmonary sepsis	13546 (5.1)	10775 (6.5)	3469 (6.9)
Cannot be classified	7106 (2.7)	6929 (4.2)	2429 (4.8)
Sepsis-related ICU admission	2118 (0.8)	2163 (1.3)	809 (1.6)
Sepsis-related death	1459 (0.6)	1786 (1.1)	715 (1.4)

Sepsis cases were identified using ICD-10 codes as defined in Supplementary file Table 1. Cases meeting the sepsis definition but lacking identifiable primary infection site were categorized as ‘Cannot be classified’.

BMI: body mass index. ICU: intensive care unit. IQR: interquartile range. MET-min/w: metabolic equivalent of task minutes per week. TDI:Townsend Deprivation Index.

Former smokers were the oldest (58.14 ± 7.70 years), followed by never smokers (55.80 ± 8.15 years) and current smokers (54.63 ± 8.14 years). A higher proportion of women was observed in current (54.2%) and former smokers (50.7%) versus never smokers (40.9%). Current smokers had the highest TDI (median = -0.41; IQR: -2.81–2.78) and lowest college degree rate (22.9%), while never smokers had the most favorable socioeconomic profile with median TDI of -2.39 (IQR: -3.78 – -0.01) and college degree (36.0%). Former smokers had the highest obesity (BMI ≥30 kg/m²) rate at 27.3% vs 22.5% for never, and 22.4% for current smokers. Physical activity was slightly higher in current smokers (2000 MET-min/week) versus former/never smokers (each 1824 MET-min/week). Current smokers had the highest prevalence of chronic respiratory disease (25.1%), chronic liver disease (5.3%), and cardiovascular disease (23.1%), while former smokers had the highest rates of hypertension (44.5%), diabetes (11.2%), chronic kidney disease (6.2%), and cancer (22.8%). Never smokers had the lowest prevalence of most comorbidities except hypertension (35.5%).

### Smoking status, pack-years, and sepsis risk

In fully adjusted Model 3, current smokers had a higher risk of sepsis (adjusted hazard ratio, AHR=1.42; 95% CI: 1.39–1.46), pulmonary sepsis (AHR=1.57; 95% CI: 1.48–1.66), and non-pulmonary sepsis (AHR=1.36; 95% CI: 1.30–1.42) (Table 2) compared with never smokers. Former smokers also had an elevated sepsis risk (AHR=1.10; 95% CI: 1.08–1.12), though the risk was lower than that among current smokers. Among ever smokers, individuals with >30 pack-years had the highest sepsis risk (AHR=1.54; 95% CI: 1.47–1.60) relative to those with ≤10 pack-years, with similar trends observed for pulmonary (AHR=1.82; 95% CI: 1.66–2.00) and non-pulmonary sepsis (AHR=1.39; 95% CI:1.30–1.48) (Supplementary file Table 4). Kaplan-Meier curves confirmed higher cumulative sepsis incidence in current smokers versus never and former smokers, as well as a stepwise increase in risk with greater pack-years (both p<0.001) (Figure 1).

**Table 2:** Associations between smoking status and sepsis outcomes: Cox proportional hazards regression analyses among UK Biobank participants free of prevalent sepsis at baseline, a secondary analysis of a prospective cohort study (N=480340) (recruitment: 2006–2010; median follow-up: 13 years)

	*n (%)*	*Incidence per 1000 people*	*HR (95% CI)*			
			*Crude model*	*Model 1*	*Model 2*	*Model 3*
**Sepsis**						
Never smoker (ref.)	25346 (9.6)	7.08	1	1	1	1
Former smoker	21963 (13.3)	9.92	1.41 (1.38–1.43)	1.18 (1.16–1.20)	1.17 (1.15–1.19)	1.10 (1.08–1.12)
Current smoker	7562 (15.0)	11.3	1.60 (1.56–1.64)	1.51 (1.47–1.55)	1.58 (1.54–1.62)	1.42 (1.39–1.46)
**Pulmonary sepsis**						
Never smoker (ref.)	4694 (1.8)	1.31	1	1	1	1
Former smoker	4259 (2.6)	1.92	1.47 (1.41–1.53)	1.23 (1.18–1.29)	1.22 (1.17–1.27)	1.14 (1.09–1.19)
Current smoker	1664 (3.3)	2.48	1.90 (1.80–2.01)	1.73 (1.63–1.83)	1.80 (1.70–1.91)	1.57 (1.48–1.66)
**Non-pulmonary sepsis**						
Never smoker (ref.)	13546 (5.1)	3.15	1	1	1	1
Former smoker	10775 (6.5)	4.02	1.28 (1.24–1.32)	1.10 (1.07–1.13)	1.10 (1.07–1.13)	1.09 (1.06–1.12)
Current smoker	3469 (6.9)	4.15	1.32 (1.27–1.38)	1.31 (1.25–1.36)	1.38 (1.32–1.44)	1.36 (1.30–1.42)
**Sepsis-related ICU admission**						
Never smoker (ref.)	2118 (0.8)	0.59	1	1	1	1
Former smoker	2163 (1.3)	0.98	1.65 (1.56–1.75)	1.33 (1.25–1.41)	1.30 (1.22–1.39)	1.21 (1.13–1.28)
Current smoker	809 (1.6)	1.21	2.05 (1.89–2.22)	1.78 (1.64–1.94)	1.89 (1.74–2.05)	1.66 (1.52–1.80)
**Sepsis-related death**						
Never smoker (ref.)	1459 (0.6)	0.41	1	1	1	1
Former smoker	1786 (1.1)	0.81	1.99 (1.86–2.14)	1.48 (1.38–1.59)	1.47 (1.37–1.58)	1.34 (1.25–1.44)
Current smoker	715 (1.4)	1.07	2.64 (2.42–2.89)	2.45 (2.23–2.69)	2.55 (2.33–2.80)	2.11 (1.92–2.32)

Model 1: adjusted for age, sex, ethnicity, education level, and Townsend deprivation index.

Model 2: adjusted as for Model 1 plus body mass index, alcohol drinking status, and physical activity level.

Model 3: adjusted as for Model 2 plus hypertension, diabetes, chronic respiratory disease, chronic liver disease, chronic renal failure, cardiovascular diseases, cancer surgery history, and medication history. For all models, p for trend <0.001.

HR: hazard ratio. ICU: intensive care unit.

**Figure 1:**
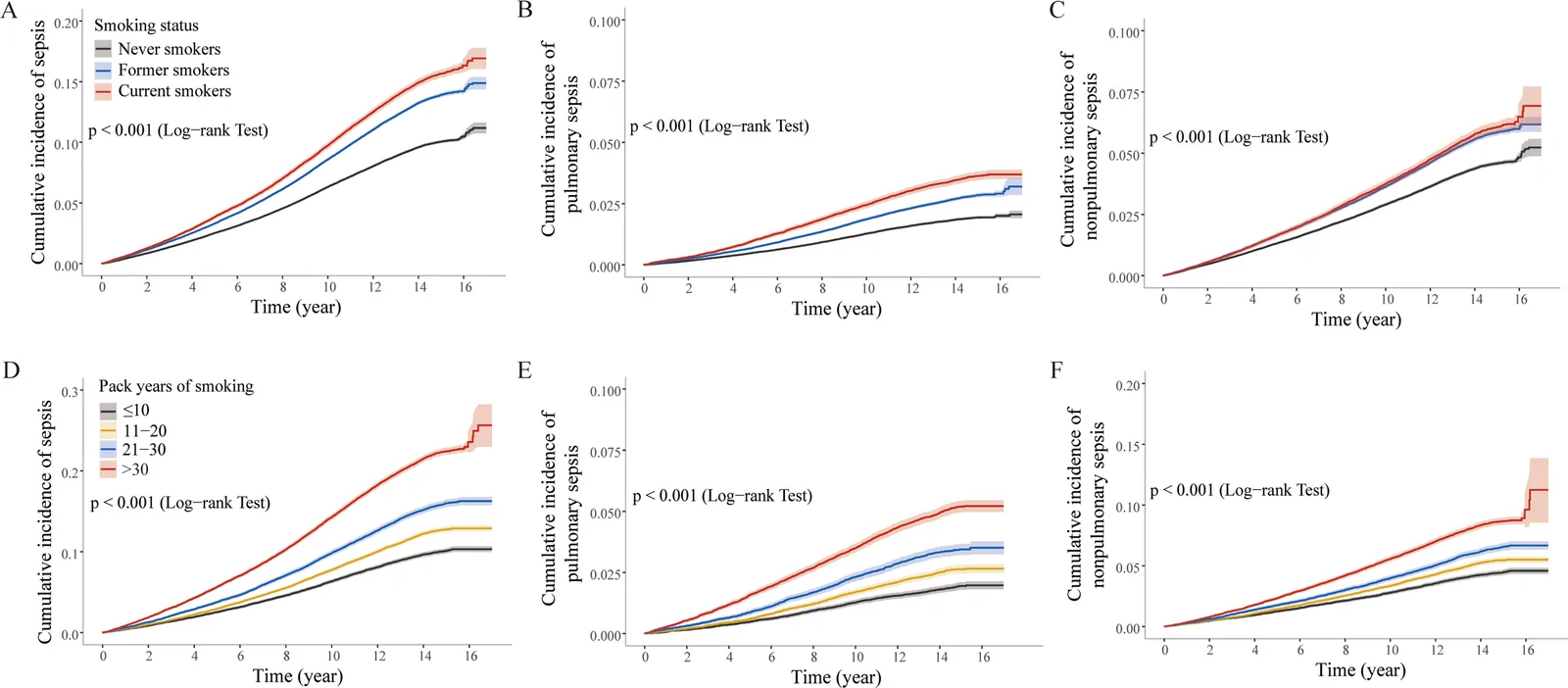
Cumulative incidence of Sepsis outcomes stratified by smoking status and smoking pack-years: Kaplan-Meier analysis of participants in the UK Biobank (N=480344) (recruitment: 2006–2010; median follow-up: 13 years)

A–C: Cumulative incidence by smoking status (never, former, current). D–F: Cumulative incidence by pack-years (≤10, 11–20, 21–30, >30). All analyses excluded participants with a prior history of sepsis at baseline. The log-rank test was performed for all comparisons (all p<0.001).

Smoking cessation had a time-dependent protective effect: former smokers who quit for >30 years had reduced sepsis risk (AHR=0.73; 95% CI: 0.70–0.76) versus those who quit for ≤10 years, with similar reductions for pulmonary (AHR=0.68; 95% CI: 0.61–0.75) and non-pulmonary sepsis (AHR=0.79; 95% CI: 0.74–0.85) (Supplementary file Table 5). Stratified analyses confirmed robust associations, with significant interactions (p<0.05) (Supplementary file Table 6): smoking-related sepsis risk was higher in those aged <60 years versus ≥60 years (current smokers: AHR=1.41 vs 1.36), stronger in men versus women (AHR=1.46 vs 1.40), and more prominent in non-obese versus obese participants (AHR=1.47 vs 1.26). Smokers without diabetes/hypertension had greater sepsis risk elevation than those with comorbidities (diabetes: p<0.001; hypertension: p=0.044). Smoking was also associated with increased sepsis-related ICU admission and mortality, with current smokers having the highest AHRs (Table 2; and Supplementary file Tables 4 and 5).

### Differential proteins and enriched pathways

The subgroup subjected to proteomic analysis shared similar baseline characteristics with the overall cohort, verifying its representativeness for the whole study population (Supplementary file Table 7). A total of 1006 proteins were found to be upregulated in patients with sepsis, with 476 and 462 proteins specifically upregulated in pulmonary and non-pulmonary sepsis, respectively (Figure 2). Among the 5 proteins with the most statistically significant differences in expression levels in sepsis and non-sepsis were Growth Differentiation Factor 15 (GDF15), WAP Four-Disulfide Core Domain 2 (WFDC2), Plasminogen Activator Urokinase Receptor (PLAUR), TNF Receptor Superfamily Member 1B (TNFRSF1B), and Insulin-Like Growth Factor Binding Protein 4 (IGFBP4). The list of the top 20 differentially expressed proteins are presented in Supplementary file Table 8.

**Figure 2:**
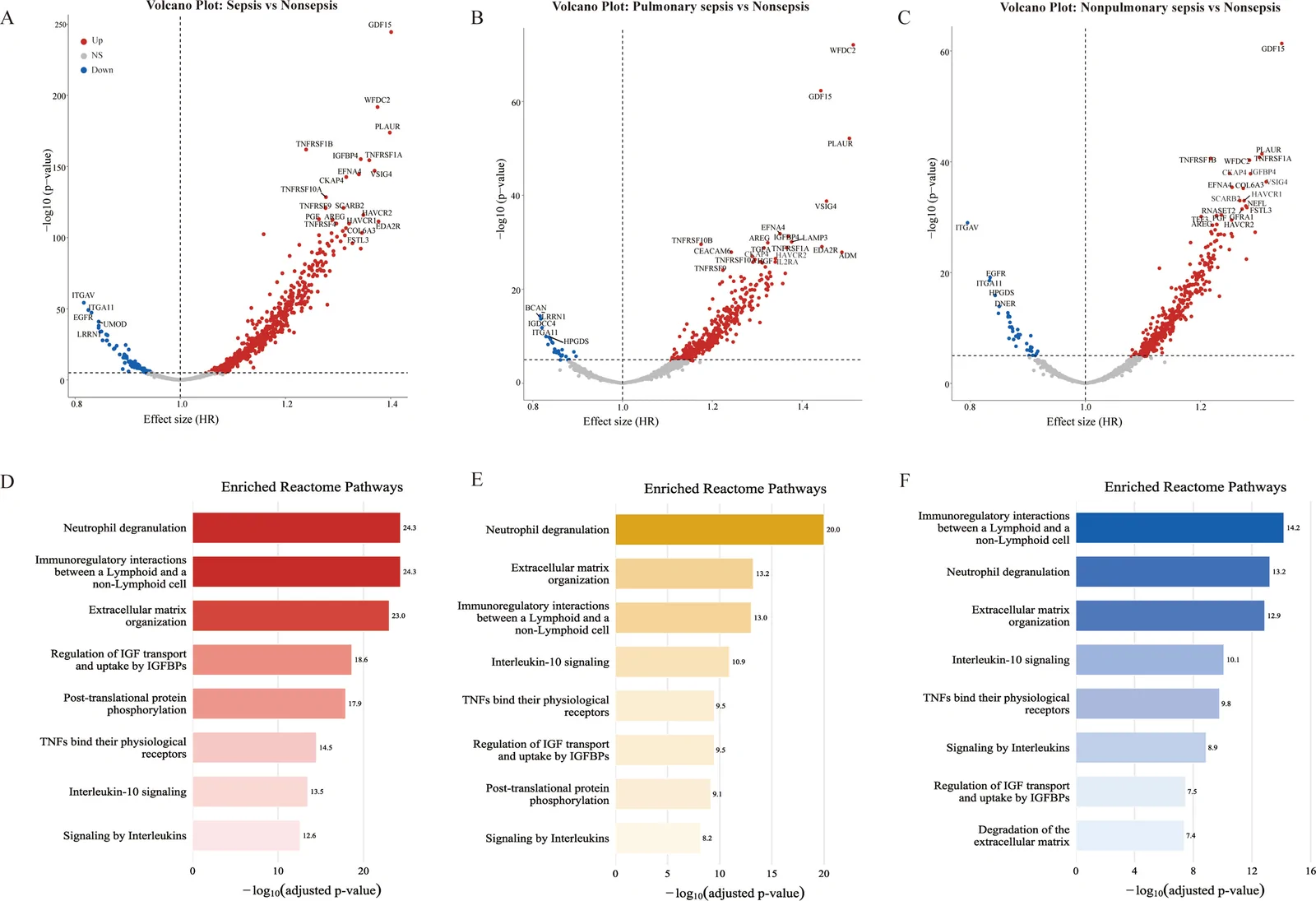
Differential expression of 2920 unique plasma proteins (Olink Explore 3072 platform) and enriched Reactome pathways among participants free of prevalent sepsis at baseline, a secondary analysis of a prospective cohort of UK Biobank proteomic participants (N=50564) (recruitment: 2006–2010; median follow-up: 13 years)

A–C: Volcano plots of differential protein expression. A: Participants with vs without sepsis. B: Participants with vs without pulmonary sepsis. C: Participants with vs without non-pulmonary sepsis. The y-axis shows the log10-adjusted p-value, and the x-axis shows the effect size as fold change. All proteins were adjusted for age. Red indicates up-regulation; blue indicates down-regulation. D–F: Over-represented Reactome pathways. D: Over-represented pathways in participants with sepsis. E: Over-represented pathways in participants with pulmonary sepsis. F: Over-represented pathways in participants with non-pulmonary sepsis. Most pathways associated with sepsis were related to inflammation and immunity, with ‘neutrophil degranulation’ being the most prominent. Several pathways were also implicated in extracellular matrix organization and in the regulation of insulin-like growth factor (IGF) bioavailability, a process primarily governed by IGF-binding proteins (IGFBP).

Based on the pathway enrichment analysis of differentially expressed proteins, biological pathways that were significantly over-represented in both pulmonary and non-pulmonary sepsis were systematically identified. Overall, the enriched pathways in both types of sepsis strongly pointed to immune and inflammatory processes, with ‘neutrophil degranulation’ being the most prominent common pathway in both groups. Additionally, pathways related to systemic immune responses, such as ‘cytokine-cytokine receptor interaction’, ‘immunoregulatory interactions between a lymphoid and a non-lymphoid cell’, ‘interleukin-10 signaling’, ‘IL-17 signaling pathway’, ‘intestinal immune network for IgA production’, and ‘extracellular matrix organization’ were commonly enriched in both types of sepsis. Notably, the pathway profiles exhibited clear infection-origin specificity: in pulmonary sepsis, the over-represented pathways were also significantly concentrated in ‘cell adhesion molecule interaction’, ‘Chemokine signaling pathway’, ‘Antimicrobial peptides’, and ‘Surfactant metabolism’, suggesting a central role of local tissue barrier disruption and inflammatory cell infiltration. In contrast, non-pulmonary sepsis further showed specific enrichment of pathways related to adaptive immune polarization (e.g. Th1 and Th2 cell differentiation), immune surveillance (e.g. natural killer cell mediated cytotoxicity), and cell fate regulation (e.g. TP53 regulates transcription of death receptors and ligands), highlighting distinct mechanisms of immune dysregulation and cellular homeostasis disruption. In addition, some pathways were related to cell surface interactions at the vascular wall and metabolism of angiotensinogen to angiotensins.

### Pathway mediation in smoking-sepsis association

Mediation analyses were employed to assess the extent to which immune and inflammatory pathways could explain the association between smoking status and sepsis risk. As shown in Table 3 and Supplementary file Table 9, the extent to which individual pathways mediated this association varied substantially by sepsis type. For pulmonary sepsis, the mediation proportion of single pathways ranged from 14.4% (e.g. antimicrobial peptides in Reactome) to 37.6% (IL-17 signaling pathway in KEGG), with general inflammatory pathways (e.g. TNF, NF-κB) showing consistent but moderate mediation. Notably, the direct effects were 1.40–1.46 for Reactome-annotated pathways and 1.24–1.30 for KEGG-annotated pathways, suggesting that smoking acts predominantly through direct damage to lung tissue. In contrast, for non-pulmonary sepsis, individual pathways explained a markedly larger and more variable proportion of the association, with mediation proportions ranging from as low as 9.4% (Th1 and Th2 cell differentiation) to as high as 69.9% (neutrophil degranulation). Pathways such as the intestinal immune network for IgA production exhibited particularly strong and consistent mediation across databases (64.3% in KEGG). Furthermore, the direct effects were much weaker (AHR=1.04–1.13). Overall, these findings indicate that smoking influences non-pulmonary sepsis risk primarily through systemic immune dysregulation mediated by specific, potent pathways, whereas its effect on pulmonary sepsis is driven more by direct local injury, with inflammation playing a significant but secondary mediating role.

**Table 3:** Mediation effects of Reactome pathways on the association between smoking status and incident pulmonary versus non-pulmonary sepsis, a secondary analysis of a prospective cohort of UK Biobank proteomic participants (N=50564) (recruitment: 2006–2010; median follow-up: 13 years)

*Pathway*	*Direct effect HR (95% CI)*	*Indirect effect HR (95% CI)*	*Total effect HR (95% CI)*	*Mediation proportion %*
**Pulmonary sepsis**				
Neutrophil degranulation	1.46 (1.29–1.64)	1.06 (1.05–1.08)	1.55 (1.37–1.75)	17.2
Extracellular matrix organization	1.41 (1.25–1.58)	1.09 (1.07–1.10)	1.54 (1.36–1.72)	23.2
Immunoregulatory interactions between a Lymphoid and a non-lymphoid cell	1.46 (1.30–1.63)	1.06 (1.05–1.07)	1.55 (1.39–1.73)	16.9
Interleukin-10 signaling	1.46 (1.29–1.65)	1.06 (1.05–1.07)	1.55 (1.37–1.75)	16.7
Signaling by interleukins	1.44 (1.28–1.62)	1.08 (1.07–1.09)	1.55 (1.38–1.76)	20.6
TNFs bind their physiological receptors	1.46 (1.28–1.62)	1.06 (1.05–1.07)	1.55 (1.37–1.72)	16.3
TNFR2 non-canonical NF-kB pathway	1.45 (1.28–1.63)	1.06 (1.05–1.07)	1.55 (1.36–1.74)	16.8
Cell surface interactions at the vascular wall	1.44 (1.27–1.61)	1.08 (1.06–1.09)	1.55 (1.36–1.74)	19.8
Metabolism of angiotensinogen to angiotensins	1.40 (1.24–1.58)	1.09 (1.08–1.11)	1.54 (1.36–1.73)	24.7
Surfactant metabolism	1.44 (1.28–1.63)	1.06 (1.05–1.08)	1.54 (1.37–1.72)	17.3
Antimicrobial peptides	1.46 (1.31–1.63)	1.05 (1.04–1.06)	1.54 (1.38–1.72)	14.4
**Non-pulmonary sepsis**				
Neutrophil degranulation	1.04 (0.99–1.10)	1.09 (1.08–1.10)	1.13 (1.07–1.19)	69.9
Extracellular matrix organization	1.06 (1.01–1.11)	1.07 (1.06–1.08)	1.13 (1.07–1.19)	56.4
Immunoregulatory interactions between a Lymphoid and a non-lymphoid cell	1.10 (1.05–1.17)	1.03 (1.02–1.04)	1.14 (1.07–1.19)	25.0
Interleukin-10 signaling	1.08 (1.02–1.14)	1.05 (1.04–1.06)	1.14 (1.07–1.19)	40.0
Signaling by interleukins	1.06 (1.01–1.12)	1.07 (1.06–1.08)	1.13 (1.07–1.20)	52.1
TNFs bind their physiological receptors	1.07 (1.02–1.12)	1.06 (1.05–1.07)	1.14 (1.08–1.19)	45.2
TNFR2 non-canonical NF-kB pathway	1.08 (1.03–1.13)	1.05 (1.04–1.06)	1.14 (1.08–1.19)	43.3
Cell surface interactions at the vascular wall	1.07 (1.01–1.13)	1.06 (1.05–1.07)	1.13 (1.08–1.20)	50.6
Metabolism of angiotensinogen to angiotensins	1.06 (1.00–1.12)	1.07 (1.06–1.08)	1.13 (1.07–1.20)	56.8
TP53 regulates transcription of death receptors and ligands	1.06 (1.00–1.11)	1.08 (1.07–1.09)	1.14 (1.08–1.19)	60.2
Constitutive signaling by aberrant PI3K in cancer	1.06 (1.00–1.11)	1.06 (1.05–1.07)	1.13 (1.06–1.19)	55.0

The sum of the percentages of indirect effects of each pathway (mediator) may not equal to the total indirect effect because of correlation and overlapping mediation effects among mediators.

HR: hazard ratio. NF-kB: nuclear factor kappa-light-chain-enhancer of activated B cells. PI3K: phosphatidylinositol 3-kinase. TNF: tumor necrosis factor. TNFR2:tumor necrosis factor receptor 2. TP53: tumor protein p53.

## Discussion

The present study found that smoking increased risk for sepsis in a pack-year dose-dependent manner, and these associations remain independent after adjusting for chronic diseases and other health behaviors. The mediating analyses showed that smoking induced systemic immune dysregulation (neutrophil degranulation, interleukin pathways, etc.) and thereby elevated overall sepsis risk, while locally impairing antimicrobial peptide and surfactant metabolism for pulmonary sepsis, and causing adaptive immune disorders for non-pulmonary sepsis.

Current smokers face the highest sepsis risk, which increases with cumulative lifetime exposure and peaks in those with >30 pack-years. This core finding aligns with prior research^20-22^. We further found a stronger association with pulmonary than non-pulmonary sepsis, linking to smoking’s direct pathogenic effects: pulmonary epithelial barrier damage, lung microbiota disruption, and local immune suppression. Subgroup analyses showed more significant smoking-sepsis associations in adults <60 years, males, non-obese individuals, and those without diabetes/hypertension. This may reflect subgroup differences in immune responses and baseline organ function. For instance, older adults or those with comorbidities already have elevated risk due to immunosenescence and multi-organ decline, leading to a ‘ceiling effect’ that attenuates smoking’s incremental impact. Additionally, smoking adversely affects sepsis prognosis. Current smokers have higher risks of ICU admission and mortality, likely due to long-term smoking-induced impairment of multi-organ reserve, which reduces compensatory capacity for sepsis-related organ failure and adversely affects outcomes.

Proteomic analysis has pinpointed key upregulated proteins (GDF15, PLAUR, TNF) as central regulators of sepsis pathogenesis, whose dysregulation is closely linked to smoking-induced immune perturbations. GDF15, an inflammation-induced hormone, plays a pivotal role in host defense against infections and sepsis^23^. PLAUR mediates extracellular matrix (ECM) degradation via plasminogen system activation, participating in septic immune microenvironment remodeling^24^. Elevated plasma TNF and its soluble receptors (sTNFR1/sTNFR2) in sepsis patients confirm its role in driving sepsis-related proliferation, inflammation, and programmed cell death^25^. These molecules converge on common pathogenic pathways of dysregulated innate immunity in sepsis. First, neutrophil degranulation serves as a key pathway. Neutrophils clear pathogens at infection sites through degranulation and extracellular trap release^26^, but smoking triggers persistent low-grade activation of this process. This leads to neutrophil dysfunction and subsequent tissue injury, rather than effective pathogen elimination. Second, the imbalance in cytokine-cytokine receptor interactions is noteworthy. TNF acts synergistically with IL-10, IFN-γ, and IL-1β signaling pathways to drive tissue injury^27^, and smoking further perturbs this cytokine network. Third, matrix metalloproteinases (MMPs) degrade ECM to facilitate immune cell recruitment to inflammatory sites. Clinical data show elevated levels of MMPs (along with TIMP-1) in sepsis patients, which correlate with SOFA-2 scores^28,29^. Smoking upregulates MMP expression and suppresses their inhibitors, disrupting tissue matrix homeostasis and accelerating sepsis-associated tissue damage. Fourth, components of tobacco smoke, such as nicotine, reach the gastrointestinal tract, altering gut microbiota, inhibiting intestinal sIgA production, and impairing mucosal immune function. This disruption promotes pathogen translocation and elevates sepsis risk^30,31^. Finally, smoking exacerbates sepsis-induced hemodynamic instability by interfering with vascular regulatory pathways and angiotensin metabolism, ultimately resulting in severe hypotension.

Although pulmonary and non-pulmonary sepsis share common pathogenic pathways, smoking induces tissue-specific mechanisms. Pulmonary sepsis is characterized by enriched antimicrobial peptide metabolism and pulmonary surfactant homeostasis pathways. Excessive antimicrobial peptides (e.g. LL-37) trigger pro-inflammatory cytokine overproduction (TNF-α, IL-1β), increasing cytokine storm severity and organ dysfunction^32^. Smoke suppresses pulmonary surfactant secretion, leading to alveolar collapse, gas exchange disorders, secondary infections, and higher mortality^33^. In contrast, non-pulmonary sepsis features adaptive immune dysregulation (Th1/Th2 imbalance). Smoke induces CD4^+^ T cell epigenetic and metabolic disorders, skewing immunity toward Th2, impairing mitochondrial function, downregulating co-stimulatory molecules and inducing T cell exhaustion via PD-1/TIM-3, causing systemic immune dysfunction and elevated sepsis risk^34^.

Importantly, while smoking cessation partially reduces sepsis risk, individuals with a smoking history still face higher risk than never smokers even after sustained abstinence. This reflects persistent immune and tissue damage from cumulative smoke exposure, linked to the differential recovery of innate and adaptive immunity. Immunologically, smoking enhances excessive post-infection innate immune activation and chronically impairs adaptive immune memory. Following cessation, innate immune abnormalities resolve relatively quickly, but smoking-induced adverse effects on adaptive immunity may persist for 10–15 years via epigenetic mechanisms (e.g. DNA methylation)^34^. These findings indicate that smoking cessation can only partially reverse immune damage, which exhibits long-term persistence, underscoring the importance of early smoking cessation to mitigate sepsis risk.

### Strengths and limitations

This study has several strengths. Drawing on the large prospective UKB cohort, we achieved adequate statistical power to rigorously examine the association between smoking and incident sepsis. Multivariate models with thorough confounder adjustment mitigated potential bias, and plasma protein mediation analyses suggest putative biological pathways that may contribute to the connection between smoking and sepsis.

Several limitations warrant discussion. Given its observational design, causal inference is not feasible, and residual confounding may remain due to unmeasured factors, including alterations in smoking status over the follow-up period. Self-reported smoking at baseline carries inherent risks of recall bias and misclassification. Circulating protein levels may not accurately mirror *in situ* tissue expression, and proteomic measurements were available only for a subset of participants. Importantly, the UKB cohort is predominantly composed of White individuals with higher socioeconomic status, which limits the generalizability of our findings to more diverse populations.

## Conclusions

Smoking is significantly correlated with sepsis risk in a pack-years-dependent manner, and smoking cessation may partially mitigate this association. Proteomic enrichment analyses suggest that immune-inflammatory dysregulation, mainly involving neutrophil degranulation, interleukin signaling, extracellular matrix remodeling, and intestinal immune pathways, may potentially mediate the smoking–sepsis relationship. These immune-inflammatory pathways yielded modest mediating effects, explaining 14.4–24.7% of the association for pulmonary sepsis and 25.0–69.9% for non-pulmonary sepsis.
