# Supplementary material for: Smoking and risk of sepsis: A secondary analysis of a UK Biobank prospective cohort with protein pathway mediation
Source: Tob Induc Dis. 2026 Oct 15;24:10.18332/tid/226617. doi: 10.18332/tid/226617 (PMC13715597; doi:10.18332/tid/226617)
Supplement: Supplementary file 1 [file TID-24-187-s001.pdf]

TID
Tobacco Induced Diseases

Supplementary file

© 2026 Wang Y. et al.

DOI:
10.18332/tid/226617

The content has been provided by the author(s) and has not been reviewed, verified, or endorsed by European Publishing. It may not have undergone peer review. The views, opinions, and recommendations expressed are solely those of the author(s) and do not necessarily reflect the position of European Publishing. European Publishing accepts no responsibility or liability for any consequences arising from the use of, or reliance on, this content.

Supplementary Materials

Supplementary Table 1. Definition of sepsis using ICD-10 codes

Diseases	ICD-10 codes
**Sepsis**	A021, A207, A217, A227, A241, A267, A282, A327, A394, A40, A41, A427, A548, B007, B377, O85, R578, A0[0-9], A19, A2[0-8], A3[0-2], A3[6-9], A4[2-9],A54,A59,A690,A691,A699,A70,A7[4-9],A8[0-1],A8[3-9],A9[2-9], B0[0-6], B0[8-9], B2[5-7], B3[3-4], B3[7-9], B4[0-6], B4[8-9], B50, B5[4-5], B5[7-9], B60, B64, B67, B9[5-7], B99, G0[0-8], H050, H602, H700, I00, I33, I3[89], I400, R02, J0[1-9], J1[0-8], J2[0-2], J36, J39[01], J8[56], K3[567], K57, K61, K63[01], K65, K750, K810, K812, K830, L0[2-4], L08, M0[01], M86, N10, N151, N30, N390, N41[023], N45, N7[0-4], N980, O030, O035, O045, O080, O23, O753, O86, O883, O91, O98, T802,T814, T826, T827, T835, T836, T845, T846, T847, T857, T880, U04
**Pulmonary Sepsis**	A36, A37, A38, A70, J0[1-9], J1[0-8], J2[0-2], J36, J39[01], J8[56], U04
**Non-pulmonary Sepsis**	A8[0-1], A8[3-9], G0[0-8], A39, I00, I33, I3[89], I400, A0[0-9], A690, K3[567], K57, K61, K63[01], K65, K750, K810, K812, K830, N10, N151, N30, N390, T827, T835, A54, A59, N41[023], N45, N7[0-4], N980, O030, O035, O045, O080, O23, O753, O86, O883, O91, O98, A3[0-2], A4[2-9], B0[0-6], B0[8-9], L0[2-4], L08, R02, M0[01], M86, T836, T845, T846

Supplementary Table 2. UK Biobank field IDs used in the analysis of smoking and sepsis

Description	Field ID
Date of attending assessment centre	53
Diagnoses - ICD10	41270
Underlying (primary) cause of death: ICD10	40001
Contributory (secondary) causes of death: ICD10	40002
Records in HES inpatient critical care dataset	41290
Date lost to follow-up	191
Smoking status	20116
Age started smoking in current smokers	3436
Age started smoking in former smokers	2867
Age stopped smoking	2897
Pack years of smoking	20161
Age at recruitment	21022
Sex	31
Ethnic	1002
Qualifications	6138
Townsend deprivation index at recruitment	22189
Body mass index	21001
Alcohol drinker status	20117
Summed MET minutes per week for all activity	22040
Date of cancer diagnosis	40005
Type of cancer: ICD10	40006
Operative procedures	41200
Medication for cholesterol, blood pressure or diabetes	6177
Protein biomarkers	1839
OLINK QC flag	144

ICD10 = International Classification of Diseases, Tenth Revision; HES = Hospital Episode Statistics; MET = Metabolic Equivalent of Task; QC = Quality Control; OLINK = OLINK Proteomics (a protein biomarker platform).

Supplementary Table 3. Associations of smoking status with sepsis outcomes: Event numbers and percentages by smoking group in a prospective cohort of 480,344 UK Biobank participants

	Never smokers<br>n = 264,030	Former smokers<br>n = 165,877	Current smokers<br>n = 50,437
Sepsis, n (%)	25,346 (9.6)	21,963 (13.2)	7,562 (15.0)
Pulmonary sepsis	4,694 (1.8)	4,259 (2.6)	1,664 (3.3)
Nonpulmonary sepsis	13,546 (5.1)	10,775 (6.5)	3,469 (6.9)
Nervous system	96 (0.0)	90 (0.1)	10 (0.0)
Circulatory system	289 (0.1)	272 (0.2)	53 (0.1)
Digestive/abdominal system	2,112 (0.8)	1,959 (1.2)	696 (1.4)
Urinary system	9,057 (3.4)	6,676 (4.0)	2,067 (4.1)
Reproductive system	521 (0.2)	454 (0.3)	142 (0.3)
Bone and joint system	574 (0.2)	371 (0.2)	98 (0.2)
Skin and soft tissue system	897 (0.3)	953 (0.6)	403 (0.8)
Cannot be classified	7,106 (2.7)	6,929 (4.2)	2,429 (4.8)
Sepsis-related ICU admission	2,118 (0.8)	2,163 (1.3)	809 (1.6)
Sepsis-related death	1,459 (0.6)	1,786 (1.1)	715 (1.4)

Sepsis cases were identified using ICD-10 codes as defined in Supplementary Table 1. Cases meeting the sepsis definition but lacking identifiable primary infection site were categorized as “Cannot be classified”.

ICU = Intensive Care Unit

Supplementary Table 4. Associations of pack years of smoking with sepsis outcomes: Cox proportional hazards regression analysis based on 480,344 participants in the UK Biobank prospective cohort

	Cases, n (%)	Incidence, per 1,000 person-years	HR (95% CI)			
			Crude model	Adjusted model 1†	Adjusted model 2‡	Adjusted model 3&
**Sepsis**						
⩽ 10	3,649 (9.6)	7.13	Reference	Reference	Reference	Reference
11–20	4,702 (12.2)	9.06	1.27 (1.22 – 1.33)	1.18 (1.14 – 1.24)	1.16 (1.11 – 1.21)	1.15 (1.10 – 1.20)
21–30	4,301 (15.3)	11.50	1.62 (1.55 – 1.33)	1.43 (1.37 – 1.50)	1.38 (1.32 – 1.44)	1.33 (1.27 – 1.39)
> 30	8,499 (21.6)	16.80	2.38 (2.29 – 2.47)	1.79 (1.72 – 1.86)	1.69 (1.62 – 1.76)	1.54 (1.47 – 1.60)
**Pulmonary sepsis**						
⩽ 10	661 (1.7)	1.29	Reference	Reference	Reference	Reference
11–20	923 (2.4)	1.78	1.47 (1.41 – 1.53)	1.23 (1.18 – 1.29)	1.22 (1.17 – 1.27)	1.14 (1.09 – 1.19)
21–30	885 (3.1)	2.37	1.90 (1.80 – 2.01)	1.73 (1.63 – 1.83)	1.80 (1.70 – 1.91)	1.57 (1.48 – 1.66)
> 30	1,811 (4.6)	3.58	2.79 (2.55 – 3.04)	2.07 (1.88 – 2.27)	1.98 (1.80 – 2.17)	1.82 (1.66 – 2.00)
**Nonpulmonary sepsis**						
⩽ 10	1,567 (4.1)	3.06	Reference	Reference	Reference	Reference
11–20	1,947 (5.0)	3.75	1.39 (1.20 – 1.60)	1.26 (1.09 – 1.45)	1.14 (1.06 – 1.22)	1.13 (1.06 – 1.21)
21–30	1,673 (5.9)	4.48	1.47 (1.37 – 1.57)	1.32 (1.23 – 1.41)	1.27 (1.18 – 1.36)	1.25 (1.16 – 1.34)
> 30	3,067 (7.8)	6.06	1.99 (1.88 – 2.12)	1.54 (1.44 – 1.64)	1.45 (1.36 – 1.55)	1.39 (1.30 – 1.48)
**Sepsis-related ICU admission**						
⩽ 10	317 (0.1)	0.62	Reference	Reference	Reference	Reference
11–20	446 (1.2)	0.86	1.65 (1.56 – 1.75)	1.33 (1.25 – 1.41)	1.22 (1.06 – 1.41)	1.20 (1.04 – 1.39)
21–30	463 (1.6)	1.24	2.00 (1.74 – 2.31)	1.69 (1.46 – 1.95)	1.60 (1.39 – 1.85)	1.53 (1.32 – 1.76)
> 30	963 (2.4)	1.90	3.08 (2.71 – 3.49)	2.12 (1.86 – 2.42)	1.96 (1.72 – 2.24)	1.74 (1.52 – 1.99)
**Sepsis-related death**						
⩽ 10	227 (0.6)	0.44	Reference	Reference	Reference	Reference
11–20	368 (1.0)	0.71	1.60 (1.36 – 1.89)	1.44 (1.22 – 1.70)	1.42 (1.20 – 1.67)	1.38 (1.17 – 1.63)
21–30	343 (1.2)	0.92	2.09 (1.76 – 2.47)	1.72 (1.46 – 2.04)	1.69 (1.42 – 1.99)	1.55 (1.31 – 1.83)

> 30	885 (2.2)	1.75	4.02 (3.47 – 4.65)	2.59 (2.63 – 3.02)	2.49 (2.14 – 2.89)	2.07 (1.78 – 2.42)

Participants were categorized by smoking pack-years into ≤10 (n=37,821), 11-20 (n=38,684), 21-30 (n=28,189), and >30 (n=39,414). *P* for trend in all models were less than 0.001.

† Adjusted for age, sex, ethnicity, educational level, and Townsend deprivation index;

‡ Additionally adjusted for body mass index, alcohol drinking status, and physical activity level;

& Additionally adjusted for hypertension, diabetes, chronic respiratory disease, chronic liver disease, chronic renal failure, cardiovascular diseases, cancer, surgery history, medication history.

HR = hazard ratio; CI = confidence interval; ICU = intensive care unit.

Supplementary Table 5. Associations of years of smoking cessation with sepsis outcomes: Cox proportional hazards regression analysis based on 480,344 participants in the UK Biobank prospective cohort

	Cases, n (%)	Incidence, per 1,000 person-years	HR (95% CI)			
			Crude model	Adjusted model 1†	Adjusted model 2‡	Adjusted model 3&
**Sepsis**						
⩽ 10	5,553 (15.3)	11.6	Reference	Reference	Reference	Reference
11–20	3,798 (14.5)	10.9	0.94 (0.90 – 0.98)	0.86 (0.83 – 0.90)	0.85 (0.82 – 0.89)	0.88 (0.85 – 0.92)
21–30	4,015 (13.5)	10.1	0.87 (0.84 – 0.91)	0.73 (0.70 – 0.77)	0.75 (0.72 – 0.78)	0.79 (0.76 – 0.82)
> 30	3,194 (14.4)	10.9	0.94 (0.84 – 0.98)	0.64 (0.61 – 0.67)	0.67 (0.64 – 0.70)	0.73 (0.70 – 0.76)
**Pulmonary sepsis**						
⩽ 10	1,132 (3.1)	2.36	Reference	Reference	Reference	Reference
11–20	736 (2.8)	2.11	0.89 (0.82 – 0.98)	0.83 (0.76 – 0.91)	0.83 (0.75 – 0.91)	0.86 (0.78 – 0.94)
21–30	756 (2.5)	1.91	0.80 (0.74 – 0.89)	0.70 (0.64 – 0.77)	0.71 (0.64 – 0.78)	0.76 (0.69 – 0.83)
> 30	581 (2.6)	1.98	0.84 (0.76 – 0.93)	0.59 (0.53 – 0.66)	0.62 (0.55 – 0.68)	0.68 (0.61 – 0.75)
**Nonpulmonary sepsis**						
⩽ 10	2,069 (5.7)	4.30	Reference	Reference	Reference	Reference
11–20	1,496 (5.7)	4.29	0.99 (0.93 – 1.06)	0.91 (0.85 – 0.97)	0.91 (0.85 – 0.97)	0.91 (0.86 – 0.98)
21–30	1,658 (5.6)	4.19	0.97 (0.91 – 1.04)	0.81 (0.76 – 0.87)	0.83 (0.77 – 0.88)	0.85 (0.79 – 0.90)
> 30	1,355 (6.1)	4.61	1.07 (0.99 – 1.44)	0.73 (0.68 – 0.78)	0.76 (0.71 – 0.82)	0.79 (0.74 – 0.85)
**Sepsis-related ICU admission**						
⩽ 10	604 (1.7)	1.26	Reference	Reference	Reference	Reference
11–20	388 (1.5)	1.11	0.88 (0.78 – 1.00)	0.82 (0.72 – 0.93)	0.81 (0.71 – 0.92)	0.84 (0.74 – 0.96)
21–30	397 (1.3)	1.00	0.79 (0.70 – 0.90)	0.69 (0.60 – 0.78)	0.70 (0.61 – 0.79)	0.76 (0.66 – 0.86)
> 30	299 (1.3)	1.02	0.80 (0.71 – 0.92)	0.56 (0.49 – 0.65)	0.60 (0.52 – 0.69)	0.67 (0.58 – 0.78)
**Sepsis-related death**						
⩽ 10	506 (1.4)	1.05	Reference	Reference	Reference	Reference
11–20	331 (1.3)	0.95	0.90 (0.78 – 1.03)	0.78 (0.68 – 0.89)	0.78 (0.67 – 0.89)	0.83 (0.72 – 0.96)

21–30	302 (1.0)	0.76	0.72 (0.63 – 0.83)	0.56 (0.49 – 0.65)	0.57 (0.50 – 0.66)	0.64 (0.55 – 0.74)
> 30	252 (1.1)	0.86	0.81 (0.70 – 0.95)	0.46 (0.40 – 0.54)	0.48 (0.41 – 0.57)	0.57 (0.48 – 0.66)

Participants were categorized by years of smoking cessation into ≤10 (n=36,331), 11-20 (n=26,234), 21-30 (n=29,692), and >30 (n=22,219). *P* for trend in all models were less than 0.001.

† Adjusted for age, sex, ethnicity, educational level, and Townsend deprivation index;

‡ Additionally adjusted for body mass index, alcohol drinking status, and physical activity level;

& Additionally adjusted for hypertension, diabetes, chronic respiratory disease, chronic liver disease, chronic renal failure, cardiovascular diseases, cancer, surgery history, medication history.

HR = hazard ratio; CI = confidence interval; ICU = intensive care unit.

Supplementary Table 6. Subgroup Analyses of the Association Between Smoking Status and Sepsis Outcomes: Cox proportional hazards regression analysis based on 480,344 participants in the UK Biobank prospective cohort

Subgroups	Smoking Status	Events/Total	Adjusted HR* (95% CI)	*P* value	*P* for trend	*P* for interaction
**Age**						< 0.001
< 60 years	Never	10,618/160,305	Reference		< 0.001	
	Former	6,397/79,892	1.08 (1.05 – 1.12)	< 0.001		
	Current	3,696/33,425	1.41 (1.36 – 1.47)	< 0.001		
≥ 60 years	Never	14,728/103,725	Reference		< 0.001	
	Former	15,566/85,985	1.14 (1.11 – 1.16)	< 0.001		
	Current	3,866/17,012	1.36 (1.31 – 1.41)	< 0.001		
**Sex**						0.004
Male	Never	14,401/156,035	Reference		< 0.001	
	Former	9,429/81,822	1.10 (1.07 – 1.13)	< 0.001		
	Current	3,324/23,080	1.46 (1.40 – 1.52)	< 0.001		
Female	Never	10,945/107,995	Reference		< 0.001	
	Former	12,534/ 84,055	1.10 (1.07 – 1.13)	< 0.001		
	Current	4,238/ 27,357	1.40 (1.35 – 1.45)	< 0.001		
**Body mass index**						< 0.001
< 30 kg/m^2	Never	17,245/204,568	Reference		< 0.001	
	Former	13,691/120,590	1.10 (1.08 – 1.14)	< 0.001		
	Current	5,474/39,150	1.47 (1.42 – 1.52)	< 0.001		
≥ 30 kg/m^2	Never	8,101/59,462	Reference		< 0.001	
	Former	8,272/45,287	1.11 (1.07 – 1.14)	< 0.001		
	Current	2,088/11,287	1.16 (1.20 – 1.32)	< 0.001		
**Alcohol drinking**						0.98
Current	Never	21,871/239,083	Reference		< 0.001	
	Former	20,120/156,636	1.09 (1.07 – 1.12)	< 0.001		
	Current	6,643/46,395	1.42 (1.38 – 1.46)	< 0.001		
Non-current	Never	3,475/24,947	Reference		< 0.001	

	Former	1,843/9,241	1.10 (1.04 – 1.17)	< 0.001		
	Current	919/4,042	1.40 (1.29 – 1.51)	< 0.001		
**Hypertension**						< 0.001
Yes	Never	12,556/93,807	Reference		< 0.001	
	Former	12,844/73,764	1.09 (1.07 – 1.13)	< 0.001		
	Current	3,831/19,532	1.35 (1.30 – 1.41)	< 0.001		
No	Never	12,790/170,223	Reference		< 0.001	
	Former	9,119/92,113	1.10 (1.07 – 1.13)	< 0.001		
	Current	3,731/30,905	1.49 (1.43 – 1.55)	< 0.001		
**Diabetes**						< 0.001
Yes	Never	3,759/20,907	Reference		< 0.001	
	Former	4,457/18,613	1.13 (1.08 – 1.18)	< 0.001		
	Current	1,346/5,797	1.24 (1.17 – 1.33)	< 0.001		
No	Never	21,587/243,123	Reference		< 0.001	
	Former	17,506/147,264	1.09 (1.07 – 1.12)	< 0.001		
	Current	6,216/44,640	1.46 (1.42 – 1.50)	< 0.001		
**Chronic respiratory disease**						< 0.001
Yes	Never	5,028/38,937	Reference		< 0.001	
	Former	5,637/30,956	1.09 (1.05 – 1.14)	< 0.001		
	Current	2,565/12,642	1.29 (1.23 – 1.36)	< 0.001		
No	Never	20,318/225,093	Reference		< 0.001	
	Former	16,326/134,921	1.10 (1.08 – 1.13)	< 0.001		
	Current	4,997/37,795	1.49 (1.44 – 1.54)	< 0.001		
**Chronic liver disease**						0.07
Yes	Never	1,362/7,888	Reference		< 0.001	
	Former	1,431/6,610	1.10 (1.02 – 1.19)	< 0.001		
	Current	600/2,651	1.26 (1.14 – 1.40)	< 0.001		
No	Never	23,984/256,142	Reference		< 0.001	

	Former	20,532/159,267	1.10 (1.08 – 1.12)	< 0.001		
	Current	6,962/47,786	1.43 (1.39 – 1.47)	< 0.001		
**Chronic renal failure**						0.004
Yes	Never	2,246/11,772	Reference		< 0.001	
	Former	2,513/10,196	1.12 (1.05 – 1.19)	< 0.001		
	Current	656/2,457	1.23 (1.13 – 1.35)	< 0.001		
No	Never	23,100/252,258	Reference		< 0.001	
	Former	19,450/155,681	1.10 (1.08 – 1.12)	< 0.001		
	Current	6,906/47,980	1.44 (1.40 – 1.48)	< 0.001		
**Cardiovascular diseases**						< 0.001
Yes	Never	7,620/48,216	Reference		< 0.001	
	Former	8,915/43,470	1.12 (1.08 – 1.15)	< 0.001		
	Current	2,950/13,657	1.30 (1.25 – 1.36)	< 0.001		
No	Never	17,726/215,814	Reference		< 0.001	
	Former	13,048/122,407	1.09 (1.07 – 1.12)	< 0.001		
	Current	4,612/36,780	1.50 (1.45 – 1.55)	< 0.001		
**Cancers**						< 0.001
Yes	Never	9,334/48,659	Reference		< 0.001	
	Former	9,047/37,759	1.13 (1.10 – 1.17)	< 0.001		
	Current	2,921/10,162	1.44 (1.38 – 1.50)	< 0.001		
No	Never	16,012/215,371	Reference		< 0.001	
	Former	12,916/128,118	1.08 (1.05 – 1.11)	< 0.001		
	Current	4,641/40,275	1.41 (1.36 – 1.46)	< 0.001		
**Surgery history**						0.06
Yes	Never	17,864/162,765	Reference		< 0.001	
	Former	16,517/111,539	1.10 (1.08 – 1.13)	< 0.001		
	Current	5,343/32,147	1.39 (1.35 – 1.44)	< 0.001		
No	Never	7,482/101,265	Reference		< 0.001	

Former	5,446/54,338	1.09 (1.05 – 1.13)	< 0.001
Current	2,219/18,290	1.48 (1.41 – 1.55)	< 0.001

*Cox proportional hazards models were comprehensively adjusted for potential confounders encompassing demographic characteristics (age, sex, ethnicity), socioeconomic factors (educational attainment, Townsend deprivation index), lifestyle factors (body mass index, alcohol drinking status, physical activity), and clinical comorbidities (hypertension, diabetes, chronic respiratory/hepatic/renal diseases, cardiovascular diseases, malignancy, surgical history, medication use). In subgroup analyses, the stratification variable was not included as a covariate in the regression models. Statistical interactions were assessed using likelihood ratio tests applied to the entire cohort. HR = hazard ratio; CI = confidence interval.

Supplementary Table 7. Baseline demographic, clinical, and lifestyle characteristics of participants in the UK Biobank prospective cohort: a comparison between the whole cohort and the proteomics subsample (recruitment: 2006–2010; median follow-up: 13 years)

Characteristics	Whole cohort	Proteomics subsample	SMD /Difference (95% CI)
Participants	480,344	50,564	
**Demographics**			
Age at entry, mean ± SD, y	56.61 ± 8.09	56.75 ± 8.21	0.03
Sex, n (%)			0.00
Men	260,937 (54.3)	27,184 (53.8)	
Women	219,407 (45.7)	23,380 (46.2)	
Ethnicity, n (%)			0.01
White	456,341 (95.0)	47,621 (94.2)	
Non-white	24,003 (5.0)	2,943 (5.8)	
Other			
TDI, median [Q1, Q3]	-2.16 (-3.65, 0.50)	-2.18 (-3.43, 0.70)	-0.07 (-0.10, -0.05)
College degree, n (%)	155,818 (32.4)	16,319 (32.3)	0.00
BMI, n (%)			0.00
< 18.5 kg/m^2	2,487 (0.5)	253 (0.5)	
18.5–24.9 kg/m^2	157,342 (32.8)	16,294 (32.2)	
25.0–29.9 kg/m^2	204,479 (42.6)	21,751 (43.0)	
≥ 30 kg/m^2	116,036 (24.2)	12,266 (24.3)	
Current drinker, n (%)	442,114 (92.0)	46,264 (91.5)	0.01
Physical activity, median [Q1, Q3], MET-min/w	1786 (815, 3560)	1782 (806, 3560)	9.00 (-0.00, 24.00)
Smoking status, n (%)			0.00
Never smokers	264,030 (55.0)	27,560 (54.5)	
Former smokers	165,877 (34.5)	17,689 (35.0)	
Current smokers	50,437 (10.50)	5,315 (10.5)	
**Comorbidities**			
Hypertension, n (%)	187,103 (39.0)	20,541 (40.6)	0.01

Diabetes, n (%)	45,317 (9.4)	5,044 (10.0)	0.01
Chronic respiratory disease, n (%)	82,535 (17.2)	8,944 (17.7)	0.00
Chronic liver disease, n (%)	17,149 (3.6)	1,854 (3.7)	0.00
Chronic renal failure, n (%)	24,425 (5.1)	3,157 (6.2)	0.02
Cardiovascular diseases, n (%)	105,343 (21.9)	11,874 (23.5)	0.01
Cancer, n (%)	96,580 (20.1)	10,217 (20.2)	0.00
**Medications**			
Blood pressure lowering medication, n (%)	52,826 (11.0)	6,029 (11.9)	0.01
Cholesterol lowering medication, n (%)	49,230 (10.3)	5,673 (11.2)	0.01
Insulin, n (%)	3,100 (0.6)	352 (0.7)	0.00
**Outcomes**			
Sepsis, n (%)	54,871 (11.4)	5,967 (11.8)	0.01
Pulmonary sepsis, n (%)	10,617 (2.2)	1,214 (2.4)	0.01
Nonpulmonary sepsis, n (%)	27,790 (5.8)	3,033 (6.0)	0.01
Cannot be classified, n (%)	16,464 (3.4)	1,720 (3.4)	0.01
Sepsis-related ICU admission, n (%)	5,090 (1.1)	627 (1.2)	0.01
Sepsis-related death, n (%)	3,960 (0.8)	455 (0.9)	0.01

TDI = Townsend deprivation index; BMI = body mass index; MET-min/w = metabolic equivalent of task minutes per week; SD = standard deviation; Q1 = first quartile; Q3 = third quartile; ICU = intensive care unit; SMD = standardized mean differences.

Sepsis cases were identified using ICD-10 codes as defined in Supplementary Table 1. Cases meeting the sepsis definition but lacking identifiable primary infection site were categorized as “Cannot be classified”.

Supplementary Table 8. Top 20 differentially expressed plasma proteins associated with overall, pulmonary and nonpulmonary sepsis (UK Biobank prospective cohort, proteomics subset N = 50,564, baseline 2006–2010)

**Sepsis**	
GDF15	Growth Differentiation Factor 15
WFDC2	WAP Four-Disulfide Core Domain 2
PLAUR	Plasminogen Activator, Urokinase Receptor
TNFRSF1B	TNF Receptor Superfamily Member 1B
IGFBP4	Insulin Like Growth Factor Binding Protein 4
TNFRSF1A	TNF Receptor Superfamily Member 1A
VSIG4	V-Set and Immunoglobulin Domain Containing 4
EFNA4	Ephrin A4
CKAP4	Cytoskeleton-Associated Protein 4
TNFRSF10A	TNF Receptor Superfamily Member 10A
TNFRSF9	TNF Receptor Superfamily Member 9
SCARB2	Scavenger Receptor Class B Member 2
HAVCR2	Hepatitis A Virus Cellular Receptor 2
PGF	Placental Growth Factor
AREG	Amphiregulin
EDA2R	Ectodysplasin A2 Receptor
TNFRSF4	TNF Receptor Superfamily Member 4
HAVCR1	Hepatitis A Virus Cellular Receptor 1
FSTL3	Follistatin Like 3
COL6A3	Collagen Type VI Alpha 3 Chain

**Pulmonary sepsis**	
WFDC2	WAP Four-Disulfide Core Domain 2
GDF15	Growth Differentiation Factor 15
PLAUR	Plasminogen Activator, Urokinase Receptor
VSIG4	V-Set And Immunoglobulin Domain Containing 4
EFNA4	Ephrin A4
TNFRSF10B	TNF Receptor Superfamily Member 10b
LAMP3	Lysosomal Associated Membrane Protein 3
IGFBP4	Insulin Like Growth Factor Binding Protein 4
AREG	Amphiregulin
TGFA	Transforming Growth Factor Alpha
EDA2R	Ectodysplasin A2 Receptor
TNFRSF1A	TNF Receptor Superfamily Member 1A
TNFRSF10A	TNF Receptor Superfamily Member 10a
CEACAM6	CEA Cell Adhesion Molecule 6
ADM	Adrenomedullin
HAVCR2	Hepatitis A Virus Cellular Receptor 2
TNFRSF9	TNF Receptor Superfamily Member 9
**Non-pulmonary sepsis**	
GDF15	Growth Differentiation Factor 15
PLAUR	Plasminogen Activator, Urokinase Receptor

TNFRSF1A	TNF Receptor Superfamily Member 1A
TNFRSF1B	TNF Receptor Superfamily Member 1B
WFDC2	WAP Four-Disulfide Core Domain 2
CKAP4	Cytoskeleton-Associated Protein 4
IGFBP4	Insulin Like Growth Factor Binding Protein 4
VSIG4	V-Set And Immunoglobulin Domain Containing 4
EFNA4	Ephrin A4
COL6A3	Collagen Type VI Alpha 3 Chain
SCARB2	Scavenger Receptor Class B Member 2
HAVCR1	Hepatitis A Virus Cellular Receptor 1
NEFL	Neurofilament Light Chain
FSTL3	Follistatin Like 3
GFRA1	GDNF Family Receptor Alpha 1
RNASET2	Ribonuclease T2
PGF	Placental Growth Factor
TFF3	Trefoil Factor 3
HAVCR2	Hepatitis A Virus Cellular Receptor 2
ITGAV	Integrin Subunit Alpha V

Supplementary Table 9. Mediation of immune-inflammatory pathways annotated by Kyoto Encyclopedia of Genes and Genomes between smoking status and onset of pulmonary versus nonpulmonary sepsis: Prospective cohort analysis of 50,564 UK Biobank individuals with proteomics data (baseline 2006–2010, median follow-up: 13 years)

Pathway	Direct effect HR (95% CI)	Indirect effect HR (95% CI)	Total effect HR (95% CI)	Mediation Proportion
**Pulmonary sepsis**				
Cytokine-cytokine receptor interaction	1.26 (1.17-1.35)	1.10 (1.09-1.11)	1.39 (1.28-1.49)	32.9
Viral protein interaction with cytokine and cytokine receptor	1.28 (1.19-1.37)	1.09 (1.08-1.10)	1.40 (1.30-1.49)	28.6
TNF signaling pathway	1.29 (1.21-1.39)	1.07 (1.06-1.08)	1.39 (1.29-1.49)	23.8
NF-kB signaling pathway	1.30 (1.22-1.39)	1.07 (1.06-1.08)	1.39 (1.30-1.49)	23.3
Intestinal immune network for IgA production	1.25 (1.15-1.33)	1.11 (1.10-1.12)	1.39 (1.28-1.48)	36.1
Th17 cell differentiation	1.29 (1.20-1.39)	1.08 (1.07-1.09)	1.39 (1.30-1.49)	26.4
IL-17 signaling pathway	1.24 (1.16-1.34)	1.12 (1.10-1.13)	1.39 (1.29-1.49)	37.6
Chemokine signaling pathway	1.30 (1.20-1.40)	1.08 (1.07-1.10)	1.41 (1.30-1.51)	27.1
**Nonpulmonary sepsis**				
Cytokine-cytokine receptor interaction	1.06 (1.00-1.11)	1.07 (1.06-1.08)	1.13 (1.06-1.19)	56.6
Viral protein interaction with cytokine and cytokine receptor	1.06 (1.01-1.12)	1.07 (1.06-1.08)	1.13 (1.08-1.19)	54.3
TNF signaling pathway	1.06 (1.01-1.12)	1.06 (1.05-1.07)	1.13 (1.07-1.19)	50.3
NF-kB signaling pathway	1.07 (1.01-1.12)	1.06 (1.05-1.07)	1.13 (1.07-1.19)	48.7
Intestinal immune network for IgA production	1.05 (1.00-1.10)	1.08 (1.07-1.09)	1.13 (1.07-1.19)	64.3
Th17 cell differentiation	1.08 (1.03-1.14)	1.05 (1.04-1.06)	1.14 (1.08-1.20)	39.0

IL-17 signaling pathway	1.06 (1.01-1.11)	1.07 (1.06-1.08)	1.13 (1.08-1.20)	56.8
Th1 and Th2 cell differentiation	1.13 (1.07-1.18)	1.01 (1.00-1.02)	1.14 (1.08-1.20)	9.4
Natural killer cell mediated cytotoxicity	1.10 (1.04-1.17)	1.03 (1.02-1.04)	1.14 (1.08-1.20)	25.8
Leukocyte transendothelial migration	1.11 (1.05-1.17)	1.03 (1.02-1.04)	1.14 (1.08-1.20)	22.8

Values are HR (95% CI) or n%. The sum of the percentages of indirect effects of each pathway (mediator) may not equal to the total indirect effect because of correlation and overlapping mediation effects among mediators.

HR = Hazard Ratio; CI = Confidence Interval; KEGG = Kyoto Encyclopedia of Genes and Genomes; TNF = Tumor Necrosis Factor; NF-kB = Nuclear Factor kappa-light-chain-enhancer of activated B cells; IgA = Immunoglobulin A; Th1 = T helper type 1 cell; Th2 = T helper type 2 cell; Th17 = T helper type 17 cell; IL-17 = Interleukin-17.
